# Navigating the facilitation journey: a qualitative, longitudinal evaluation of ‘Eat Walk Engage’ novice and experienced facilitators

**DOI:** 10.1186/s12913-023-10116-3

**Published:** 2023-10-20

**Authors:** Gillian Harvey, Sarah Collyer, Prue McRae, Sally E. Barrimore, Camey Demmitt, Karen Lee-Steere, Bernadette Nolan, Alison M. Mudge

**Affiliations:** 1 Caring Futures Institute, Flinders University, Adelaide, Australia; 2 Australian Centre for Health Services Innovation, Queensland University of Technology, Brisbane, Australia; 3 Royal Brisbane and Women’s Hospital Department of Internal Medicine and Aged Care, Brisbane, Australia; 4 Queensland University of Technology Institute of Health and Biomedical Innovation, Brisbane, Australia; 5 The Prince Charles Hospital, Brisbane, Australia; 6 Caboolture Hospital, Caboolture, Queensland Australia; 7 University of Queensland Faculty of Health and Behavioural Sciences, Brisbane, Australia; 8 Queensland Health, Birtinya, Australia; 9 University of Queensland Faculty of Medicine, Brisbane, Australia

**Keywords:** i-PARIHS, Facilitation, Implementation, Facilitator development

## Abstract

**Background:**

The Promoting Action on Research Implementation in Health Services (PARIHS) and integrated-PARIHS (i-PARIHS) frameworks position facilitation as an overarching strategy to enable implementation. In the revised i-PARIHS framework, facilitation is operationalised through a multi-level model with novice, experienced and expert facilitators working together in a network structure to build facilitation knowledge and skills along a continuum. To date, there has been limited evaluation of this facilitation model in practice, which is the aim of the study reported here.

**Methods:**

A descriptive, qualitative longitudinal study was undertaken to track a team of four novice and two experienced facilitators involved in facilitating the implementation of an intervention known as ‘Eat Walk Engage’ to improve multidisciplinary team delivery of age-friendly care principles in hospital. Over an 18-month period, repeat interviews were conducted to explore the learning, development, and evolving roles of novice facilitators and the roles of the experienced facilitators in providing support and mentoring. Interview data were analysed using a descriptive qualitative approach and findings were interpreted in collaboration with the participating facilitators.

**Results:**

The findings demonstrated experiential learning in both the novice and experienced facilitator groups as they enacted their roles in practice. The novice facilitators progressively transitioned to becoming more experienced facilitators and the experienced facilitators became increasingly expert, in line with the i-PARIHS concept of a facilitation journey from novice to expert. Strategies to support this development included a staggered approach to learning, regular meetings between the experienced and novice facilitators, reflective writing and informal peer support and networking. However, the roles were not without challenge and these challenges changed over time, from a more specific focus on the demands of the facilitator role to concerns about embedding and sustaining improvements in practice.

**Conclusions:**

Within a network of peers and a mentored relationship with more experienced facilitators, individuals who are new to an implementation facilitator role can transition along a continuum to become experienced facilitators. Building implementation facilitation capability in this way takes time and requires tailored support and mentorship using a mix of structured and flexible approaches incorporating opportunities for reflection to support individual and group learning.

## Contribution to the literature

- The i-PARIHS framework proposes a networked model of novice, experienced and expert facilitators. This study is one of the first empirical evaluations of the i-PARIHS facilitation model in practice.
- The study demonstrates that a facilitation network, where less experienced facilitators have a mentored relationship with more experienced facilitators, supports experiential learning and development of facilitation ‘know-how’. In turn, facilitator roles can evolve over time, from a specific project to a wider organisational focus.
- Facilitation is a dynamic process and journey. A combination of formal and informal strategies helps to structure and support facilitator learning and development.
- Facilitation is a resource-intensive implementation strategy. Future research could investigate when and how facilitation produces an acceptable return on investment.

## Background

Facilitation is positioned as an overarching implementation strategy, with a focus on enabling others to implement innovations and improvements in practice, as opposed to telling, directing, or using persuasion to encourage change [1]. It encompasses facilitator roles and facilitation strategies that are aligned with a philosophy of enabling and empowering others to act. It has been adopted as an approach to implementation across a broad range of initiatives in health care in different settings, different countries and for different interventions [2]. These facilitation approaches are often underpinned by the Promoting Action on Research Implementation in Health Services (PARIHS) and integrated-PARIHS frameworks [3–5], which explicitly position facilitation as the active agent for implementation, unlike many other implementation theories, frameworks, and models. Facilitators can be external or internal to the organisational setting and use a wide range of discrete implementation strategies, combining and tailoring these to the specific implementation initiative and context in which they are working [6]. Enacting the role involves a combination of so-called ‘hard and soft’ skills, including project management, problem solving, stakeholder engagement, relationship building, communication, teamwork, negotiation, and evaluation (see Table 1).

**Table 1:** Enacting facilitation and facilitator roles

Stetler et al. (2006) [7]	Dogherty et al. (2010) [6]	Bidassie et al. (2015) [8]	Kitson & Harvey (2016) [9]	Baloh et al. (2018) [10]	Ritchie et al. (2021) [11]
External facilitation	Internal & external facilitation	External facilitation	Internal & external facilitation	Internal facilitation	Internal & external facilitation
• Interactive problem solving and support: - Problem identification and resolution - Communication and formative use of data - Supportive relationship	• Planning for change • Leading and managing • Monitoring progress and ongoing implementation • Evaluating change	• Communication • Relationship building • Methods training • Monitoring performance over time • Facilitating team-based problem solving	• Project management and improvement skills: - Clarifying & engaging - Assessing & measuring - Action & implementation - Reviewing & sharing Team and process skills Influencing and negotiating skills	• Leadership to create and manage the planned change • Buy-in, ensuring support & engagement • Customisation, tailoring to specific organisational needs and circumstances • Accountability to sustain implementation	• Communication - Building relationships & creating a supportive environment - Changing the system of care - Transferring knowledge & skills - Planning & leading change efforts - Assessing people, processes, and outcomes

### Facilitation in the PARIHS and i-PARIHS frameworks

The original PARIHS framework proposed that the successful implementation of evidence into practice was a function of the interplay between the nature of the evidence to be implemented, the context where implementation was occurring and the way in which the process was facilitated [3]. The development of PARIHS was informed by experiential knowledge of working as facilitators of quality improvement and practice development, where the emphasis was on embedding change at a local level through enabling participation, engagement, and ownership of the changes to be implemented [3].

In response to application, evaluation, and critiques of the PARIHS framework over several years [12–15], the framework was revised to the i-PARIHS framework, with a more explicit focus on operationalising the facilitation construct [5, 16]. This involved a more detailed description of facilitation roles and functions and a facilitator’s ‘toolkit’ to support facilitation activities at the level of an implementation project [16]. i-PARIHS recognises the complex nature of facilitation, encompassing a broad range of discrete implementation strategies (including, for example, interactive education, audit and feedback, quality improvement methods, reminder systems), and identifies the experiential development of knowledge and skills to fulfil the role. It proposes a network of facilitators with a continuum of expertise from novice to experienced and expert facilitators whereby facilitators who are new to the role are developed, mentored, and supported by facilitators who are more experienced [9]. As such, novice facilitators embark on an experiential journey to build their skills, confidence, and ability in the role, typically starting off facilitating a defined implementation project within a local setting. As their knowledge, skills, and confidence build, they move to work at a wider organisational level and may develop, mentor, and support a new set of novice facilitators, in turn guided and supported by an expert facilitator, who is generally external to the organisation. In this way, a network of facilitators is established, and facilitation capability is expanded within an organisation.

**Fig. 1:**
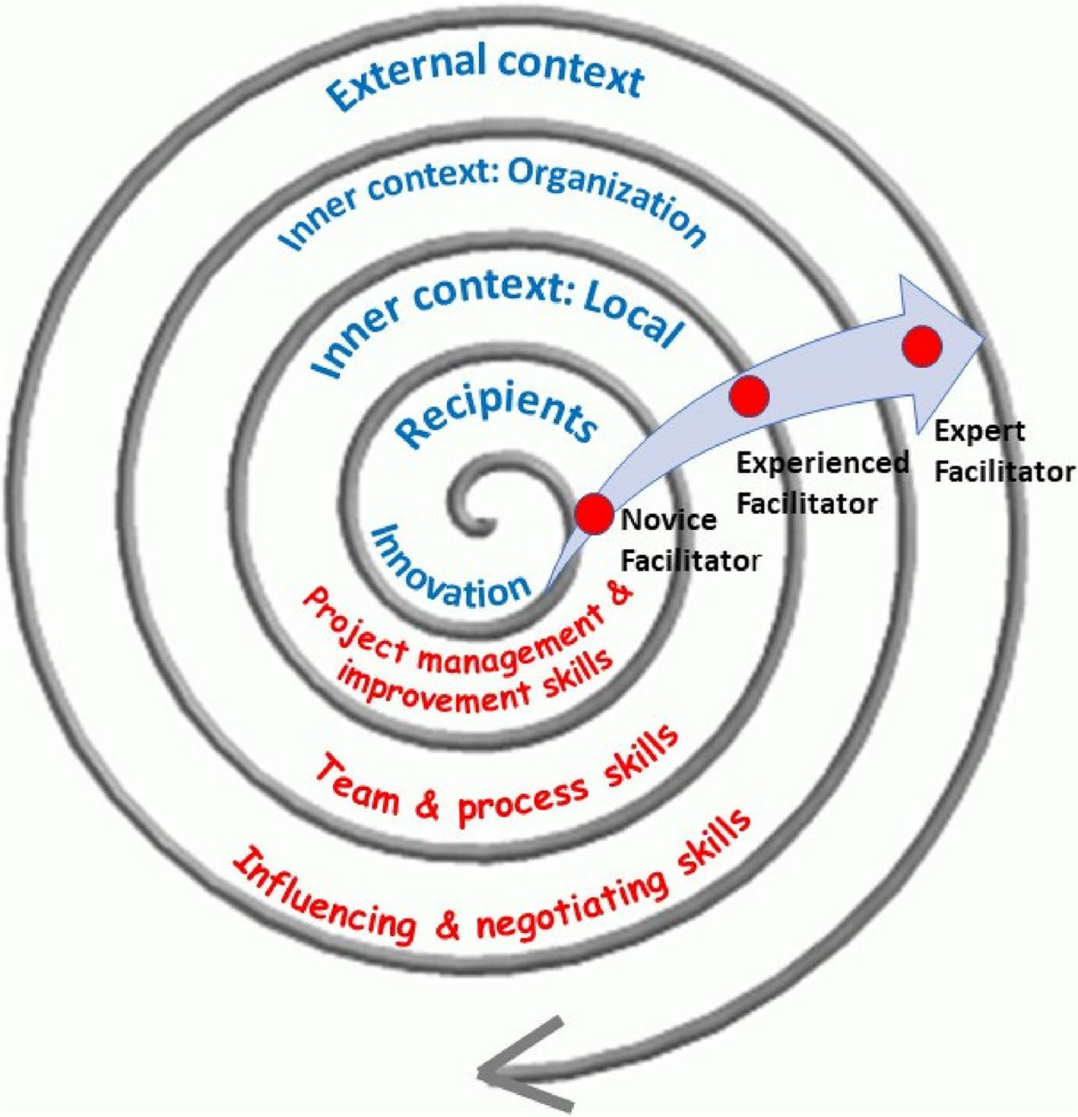
Levels of facilitation focus and activity

As transition along the facilitation continuum occurs, additional skills and knowledge are required, such as influencing and negotiating skills to address organisational level barriers to implementation (for example, time for clinical staff to participate in implementation projects or educational programs to support acquisition of new knowledge and skills) and political skills to understand the wider policy environment (with associated levers or mandates for change) in which implementation is taking place. The i-PARIHS framework illustrates this multi-level model of facilitation within a spiral of contextual levels, each exerting different influences on implementation and requiring a different focus from a facilitator perspective (see Fig. 1). Table 2 summarises the typical role and activities that the different facilitator roles would be undertaking.

**Table 2:** Levels of facilitation

	**Facilitation focus and activity**
Novice Facilitator	Working under the supervision and mentorship of an experienced or expert facilitator Focusing on an implementation project at a local level: ◦ Clarifying the problem ◦ Identifying and engaging stakeholders ◦ Reviewing evidence to inform problem solution ◦ Assessing barriers and enablers of implementation ◦ Developing and tailoring implementation strategies ◦ Building effective team-working ◦ Monitoring and communicating progress ◦ Planning for sustainability Reflecting on facilitation and networking with other facilitators to build knowledge and skills
Experienced Facilitator	Working under the supervision and mentorship of an expert facilitator In-depth understanding and knowledge of the organisation or organisations they are working with Experienced and knowledgeable in applying implementation theories and frameworks Awareness of competing tensions around implementation and how to manage these Able to assess system-wide activities and influence actions Awareness of wider contextual issues and confident in terms of managing boundaries and political tensions
Expert Facilitator	Expert facilitator operating as a guide and mentor to other facilitators Mentoring and coaching experienced and novice facilitators Coordinating facilitation networks Enabling sustainability and scale-up Informing and influencing policy to support implementation Applying, developing and/or testing theories of implementation and facilitation Evaluating implementation interventions and generating new knowledge Developing and refining learning materials and mentoring processes

There has been limited evaluation of the facilitation journey proposed in the i-PARIHS framework, particularly in terms of how facilitators acquire skills and build expertise over time. As illustrated in Table 1, previous studies have identified the types of activities that facilitators undertake and related knowledge and skill requirements. However, having a taxonomy of facilitation activities is not the same as being able to apply them in practice. As Ritchie and colleagues note, this requires tacit knowledge and processes in place to support experiential learning [11]. In their recently published study, informed by the i-PARIHS framework, the authors examined an external expert facilitator working with two novice facilitators and identified 21 techniques that were used by the expert to transfer implementation facilitation skills. These encompassed a range of cognitive, psychosocial, self-learning and structural learning supports, encompassing both active (providing information, modelling, and coaching) and participatory methods [11].

The current study aims to build further understanding of the development of facilitation ‘know-how’ by following the longitudinal journeys of four novice facilitators over an 18-month period. The specific objectives of the paper are to:

i. Assess the learning, development, and evolving role of novice facilitators in the implementation of a complex inpatient intervention ‘Eat Walk Engage’.
ii. Explore the roles of the two experienced facilitators supporting and mentoring the novice facilitators.
iii. Compare the theoretical model of facilitation (informed by i-PARIHS) with what happened in practice.

### The eat walk engage intervention

Eat Walk Engage (EWE) is a hospital ward-based programme developed to improve multidisciplinary team delivery of age-friendly care principles (adequate nutrition and hydration, early regular mobility, and meaningful cognitive and social activities) to reduce hospital-associated complications in older inpatients [17, 18]. The implementation component of EWE is underpinned by the i-PARIHS framework and involves a trained novice site facilitator working with a local multidisciplinary work group to implement the intervention.

Following initial pilot testing and refinement of EWE, a hybrid effectiveness-implementation cluster randomised trial—the Collaborative for Hospitalized Elders: Reducing the Impact of Stays in Hospital (CHERISH)—was undertaken between October 2016 and May 2017 to evaluate implementation of EWE in four hospitals. Implementation involved a local site (novice) facilitator (one for each hospital) establishing and facilitating a ward-based multidisciplinary work group to implement EWE within their own setting. The novice facilitators were allocated 16 h per week to undertake the role and received support from 2 experienced facilitators who had been responsible for the development and prior testing of EWE. These experienced facilitators (AM, PM) were clinician researchers (with backgrounds in general medicine and physiotherapy) employed within one of the hospitals and were principal investigators for the overall evaluation study. The experienced facilitators' time commitment totalled 24 h per week to provide project management, external facilitation across the project sites, and mentoring and support of the novice facilitators. Initial group training was delivered by the experienced facilitators over 4 half-days, guided by the i-PARIHS facilitator’s toolkit [16, 19]. Thereafter, the experienced and novice facilitators all met together for monthly mentoring sessions in the form of half-day face-to-face peer group meetings. Informal individual telephone and email support was provided on an as needed basis between meetings. The experienced facilitators also visited each ward that was implementing EWE before and during the implementation period to meet key stakeholders and participate, alongside the novice facilitator, in the multidisciplinary work group meetings. This included providing one on one support as required for particular activities (e.g. reflecting on local context, undertaking observational audits). External advice for the experienced facilitators was provided via a project implementation steering group, which included other experienced and expert facilitators (GH, IB, AY), along with a consumer representative.

Details of the study and its effect of a significant reduction in hospital-associated delirium have been published previously [20, 21]. The accompanying process evaluation paper [22] documents how the implementation of EWE occurred across the four different hospital settings and identifies the multiple strategies that novice facilitators used to facilitate 45 discrete improvements in care. This paper focuses on the experiences of the novice and experienced facilitators, including their perceived learning and development, as they enacted their roles to support the implementation of EWE.

## Methods

A descriptive qualitative longitudinal design was applied to track the novice and experienced facilitators supporting the implementation of EWE.

### Participants and data collection

Participants were four novice site facilitators (NFs 1–4) and two experienced facilitators (EF1 and EF2). The novice facilitators were mid-level health professionals from allied health (dietetics and occupational therapy) and nursing backgrounds, who were recruited from within the hospital. Most had previous involvement in quality improvement, patient safety and/or clinical guideline initiatives and shared the part-time facilitator role with another clinical or project role within the same hospital, which was separate from the CHERISH study. The experienced facilitators also had previous experience in implementation, quality improvement and facilitation. All 6 facilitators (novice and experienced) were female. Semi-structured interviews with novice (*n* = 4) and expert (*n* = 2) facilitators were conducted (by GH) at 3 time points over an 18-month period: early, mid, and post-implementation (Table 3). Each interview lasted 45–60 min and was conducted face to face. Participants were offered the opportunity to review their individual transcription for verification purposes. Verbal consent was given, and interviews were recorded and transcribed verbatim.

**Table 3:** Summary of data sources and collection

Facilitator group	0–3 months (R1)	6–9 months (R2)	15–18 months (R3)	TOTAL NUMBER OF INTERVIEWS
Novice facilitators (4)	4	4	4	12
Expert facilitators (2)	0	2	1^a^	3
**TOTAL NUMBER OF INTERVIEWS**	4	6	5	15

^a^2 experienced facilitators were interviewed together

### Data analysis

Transcripts were analysed using a descriptive qualitative approach [23]. Transcripts were read multiple times by two of the authors, one who was a member of the implementation steering group (GH) and a second researcher who was external to the implementation study and process evaluation (SC). Content analysis was used to code data, group codes into categories and subsequently identify four major themes [24]. Analysis was conducted using MAXQDA Analytics Pro 2020 20.4.0. The team of novice and experienced facilitators were engaged in reviewing and interpreting the emergent findings.

### Ethical approval

The evaluation was approved by the Human Research Ethics Committee of the Royal Brisbane and Women’s Hospital (HREC15/QRBW/95) and Queensland University of Technology. Interviews were audio recorded with informed consent.

## Results

Across the 18-month period of following the novice facilitators, changes were apparent in key aspects of the role, reflecting experiential learning and progression along the facilitation journey. These changes are discussed in relation to 4 themes: enactment of the facilitator role; learning about the role; strategies to support learning and development; and challenges encountered. Key findings relating to these themes at the different time-points of study are summarised in Table 4.

**Table 4:**
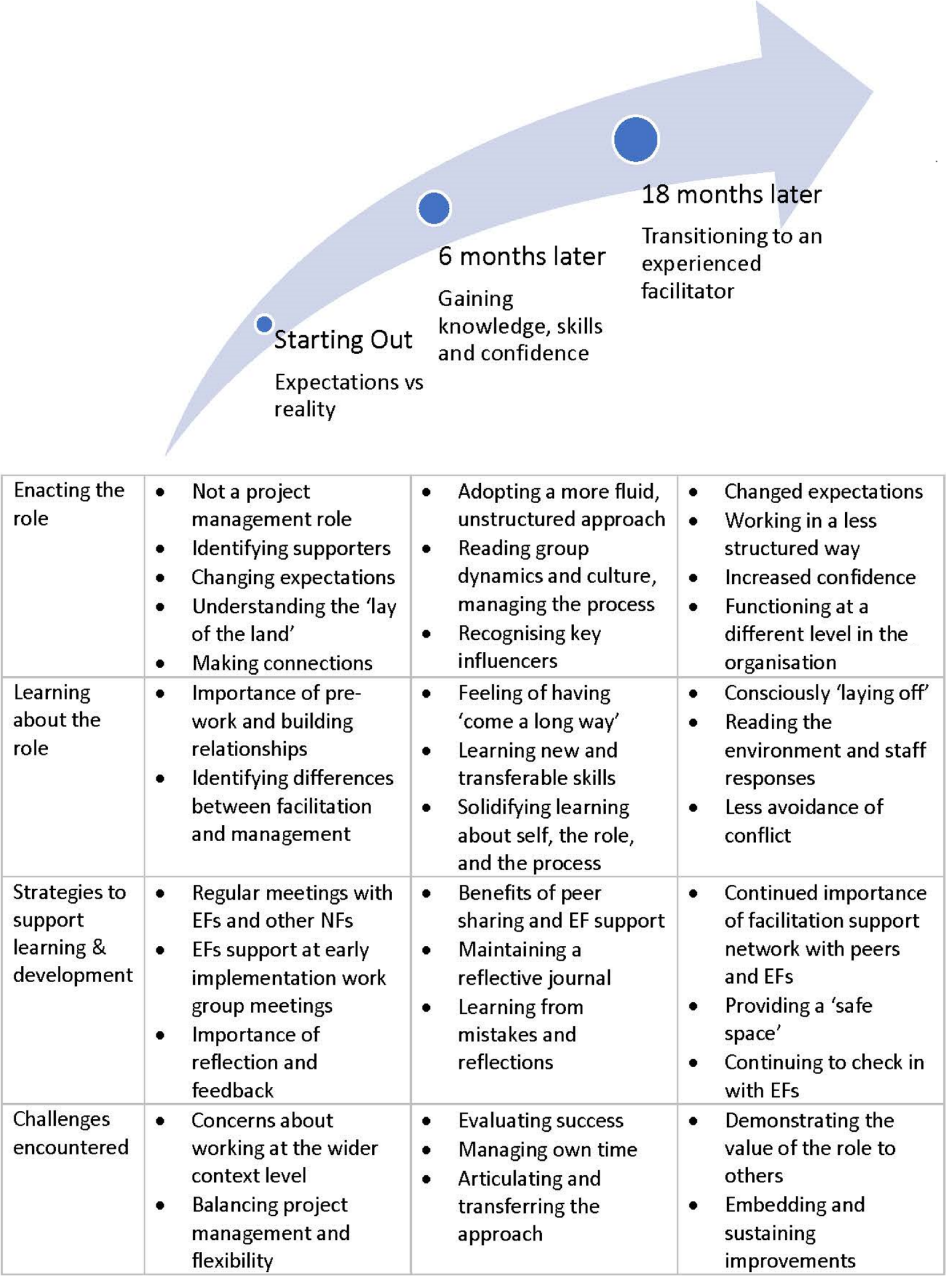
Novice facilitator journeys over an 18 month-period

### Enacting the role

Having come into the facilitator role from mid-level allied health and nursing backgrounds, often with some experience in quality improvement, clinical guideline and patient safety initiatives, expectations of what the facilitator role would involve did not always match with the reality. For some novice facilitators, this was influenced by previous experience of project management and an expectation that facilitation would be a project management type role.

*I’ve been involved in previous [health service] projects and projects are very different from the way in which this program is run and the principles behind it. I think going into it I probably had expected it to be more project like and utilising probably more concrete project management skills and for some more clearly defined timeframes for key markers of accomplishment, I guess you’d say, but I think in fact that mindset has changed quite considerably.* [NF4]

Once in the facilitator role, expectations began to change as the novice facilitators recognised the importance of a more organic approach empowering others to take ownership of the implementation process.

*I guess my view has changed and something that has changed in my thinking is I’m not the strategy and I’m not going to be fixing things, I’m building capacity on the wards. …. so working out what other people need to do or who to talk to, to get something happening rather than telling people what they need to do, if that makes sense?* [NF3]

This involved a considerable amount of initial work to build connections and relationships, identify potential supporters and generally understand the “*lay of the land*” [NF3]. Novice facilitators engaged in a range of activities to support this groundwork, including one-on-one meetings with key individuals, informal coffee catch-ups and spending time in the implementation context to observe and shadow typical clinical activity.

Around 6 months into the role, novice facilitators appeared more confident and comfortable with the flexible nature of the facilitation role, compared to what they had originally expected and could see that it would lead to more sustainable change in the longer-term.

*… I’m able to kind of step away from that project manage-y kind of feeling about … ticking off this and this and this and that and ‘we’ve done that and we’ve done that’. You have to have an element, I suppose, of some organisation in with it but it’s more flexible and it can be fluid and I’m trying to put it back to others as well, to take some of that ownership* [NF1]

However, the extent to which they were facilitating ward-based work group meetings without one of the experienced facilitators present was variable. For example, one novice facilitator had not yet facilitated any meetings on her own, whereas another had done all but one without the presence of the experienced facilitator. From the experienced facilitator perspective, the novice facilitators had required more support than originally anticipated, including training in interpreting and presenting data, and on-site meeting support.

*I feel like we’re investing a lot in facilitator support but they’re responding really well to that and I think if we didn’t they would be at sea so I think it’s been critical to have that really quite - you know, a lot of hand holding early on and then giving them a lot more autonomy as time has gone by.* [EF2]

As the novice facilitators became increasingly confident, the experienced facilitators were able to move towards providing less directive input.

*At the beginning, it was much more directive, or content driven, and then it very rapidly became – you know, so the last four meetings have really not been us talking, it’s been them talking.* [EF1]

By the completion of the study, the novice facilitators reflected on how their expectations of a facilitator role had changed significantly over time, in terms of becoming less structured, more patient with the pace of progress and recognising that they needed to be in it for the long term to achieve the desired changes in care. As novice facilitators grew in confidence, it was also clear to see that they were moving from working solely at the inner context level to greater involvement at the organisational context level, building new relationships and engaging in wider discussions, partly due to the deliberate strategy of the experienced facilitators.

*We really pushed, and supported, and mentored, a lot [ into the] organisation context rather than the ward context – challenging, and actually needed quite a lot of support, but actually managed really well.* [EF1]

### Learning about the role

As they started out in the role, novice facilitators began to develop insights into what the role involved and the implications in terms of their own learning and development. For some, this involved becoming clearer about the distinction between facilitation and management:

*I guess probably the biggest area I will be learning or getting some more knowledge and skills and learning I think will be that concept of facilitation versus sort of management ….. I think that’s going to be really interesting and really good, to have those skills and learn those skills … but I can foresee initially it’s going to be hard to bite my tongue, so to speak* [NF2]

Similarly, other novice facilitators talked about accepting the need for greater flexibility and patience in guiding or nudging people along “*so that they think they have done it all by themselves*” [NF1]. One novice facilitator described this as requiring a degree of intuition, accompanied by reflection to build skills and confidence.

*I think you have to be a little bit intuitive to some degree to be able to go with it but still be going - having that feedback loop for yourself and for your own professional practice that you know you’re going in the right direction because that’s where the confidence needs to come from as well because it is very organic...* [NF4]

Whilst the initial training had helped novice facilitators to prepare for the role, they recognised that this would probably need to be re-visited once implementation projects were underway and issues and challenges presented in real-time. In terms of their role within the wider project, novice facilitators described themselves as working at the inner context level – *the middle of the i-PARIHS spiral* [NF3] – and relying on the experienced facilitators to function at the wider organizational context level.

Novice facilitators described learning about themselves, the facilitator role, and the facilitation process over time. After the first 6 months or so, they recognised a range of improvements in terms of their skills and knowledge, including how to read group dynamics more accurately, managing group processes and conflict, learning how to pace activities, negotiating without coming across as too directive, knowing what facilitation approaches to use when and the importance of engaging with key stakeholders such as the Nurse Unit Manager.

*I feel like I’ve come a long way from six months ago, mostly I think just in terms of being able to engage at a variety of different levels and being a little bit more sensitive to the needs of the various groups on the ward. …. I have a better understanding of how to go in softly I guess and, yeah I guess use different techniques for different individuals to gain and build relationships and get their opinions and try and see where their aims for the ward would kind of drive things.* [NF4]

Evidence of the enabling focus of facilitation was clear in the way that one of the novice facilitators described the skill of “*giving that sense of accomplishment back to the staff*” [NF1], citing an example where they had helped to get the meal delivery times to the ward changed.

*I think one of the things to learn is not taking the glory for this, so presenting back at the next meeting and …. saying ‘this is what the team came up with. This is what the team, as a team, was able to influence and change. This is the outcome’.* [NF1]

Encouraging the identified ward champions and team members to take ownership of implementation became more apparent as the novice facilitators gained knowledge, skills, and confidence in the role and moved away from a directive, project management style of affecting change.

*The way they interact at their working group meetings is much more ‘tell me what you think. Tell me how you think this would work’ rather than ‘you need to do this’.* [EF1]

### Strategies to support learning and development

Both formal and informal strategies were in place to support learning and development of the novice facilitators, including monthly face-to-face meetings for the 4 novice facilitators with the experienced facilitators and weekly telephone support. The way in which the experienced facilitators worked with the novice facilitators was *“practice-based support”* [NF4], which was helpful and pragmatic.

*I would say like one of the things I really have liked about working with [the two experienced facilitators] is that …. they’re a bit more down to earth where I tend to really get - that perfectionist in me comes out …. I guess it helps to decrease my nervousness around that because they are pretty easy to work with and they have a bit more of a laid-back style. …. You know, they’re realistic so I think that that’s been really good and helpful so from that point of view it’s been wonderful.* [NF1]

The experienced facilitators deliberately adopted this approach, based on their experience of an earlier pilot study of the EWE intervention. This informed their decision to adopt a staggered approach to learning rather than conducting a period of intense training at the start of the project.

*We started with some nice, structured tasks ‘okay, go and interview ten patients and come back and put it together’ and how would we display it? … then they were starting to get to know the environment and then we started introducing the key concepts of facilitation, like engaging and measuring and assessing and things. That worked much better.* [EF2]

Alongside the more structured learning mechanisms, a growing sense of teamwork and peer support amongst the novice facilitators was apparent and was helping to building skills and confidence.

*We usually kind of send out emails to each other like ‘help’ if we have a problem and that’s been really good. I think that’s really nice, to have the other facilitators around the same stage as where I’m at because you can bounce ideas off each other or … just knowing you’re not the only one that has that question.* [NF1]

These structured and unstructured opportunities for reflection and learning continued to be centrally important throughout the 18 months of the study, helping the novice facilitators to extend and solidify their learning. Novice facilitators referred to the benefits of learning from hearing each other’s examples of things that had worked well or not so well. Additionally, the novice facilitators had reflective journals (*the little pink book –* NF2) which they used in different ways to record thoughts, reminders, notes from mentoring meetings or key events.

*I probably don’t use it as much as some of them do. I don’t write a daily entry, or whatever, I tend to write when I have a key interaction, so a real leverage moment, I guess you’d call it.* [NF4]

*I wasn’t sure how to use this book initially but what I’ve changed it into is each week, on a Thursday or Friday, I just write down the three big -- couple of big things of what’s happened that week or what I’ve spent my time doing that week and then things to follow up for the next week or query people to talk to.* [NF3]

Regular novice facilitator and experienced facilitator meetings were viewed by both parties as critically important, providing a safe space for bidirectional learning, sharing, support, mentoring and debriefing. Although the experienced facilitators “*didn’t anticipate quite so much face to face, peer to peer support”,* they believed it was an *“incredibly valuable”* component of the facilitation model [EF2].

*One morning a month we spend three hours all together and we obviously learn more about their context and their recipients […] we learn all sorts of things from them and can reflect on how we’ve done things in different settings […] I think we’re becoming much better facilitators just by watching other people learn as well.* [EF2]

### Challenges encountered

Starting out in the facilitator role, the types of challenges encountered by the novice facilitators included getting the local implementation working groups established, setting up communication structures and balancing flexibility and project management. Additionally, for some of the novice facilitators, they had to juggle the half-time novice facilitator role with another position, such as a clinical or project responsibility, which meant learning how to manage their time effectively. Concerns were also raised about the need to engage with staff beyond the immediate work group they were responsible for facilitating, particularly where this involved communicating with senior medical staff.

*I’ve been getting quite a few questions from some consultants [about the study design]. I feel like my skills are improving in responding to them and not being scared of them when they go … ‘Why are you doing this study like this?’ I feel a bit more confident now and I think that’ll only improve now with [experienced facilitator’s] backing* [NF3]

Challenges continued to be faced over time, although they changed in nature as the focus shifted from how the novice facilitator carried out the role to concerns about embedding and sustaining improvements in practice. Concerns related to issues such as staff turnover, maintaining momentum, demonstrating achievements, and convincing others of the value of facilitator roles to support implementation. Novice facilitators commented on a need for ongoing facilitation support as progress appeared to stall or fall back when they were less present on the ward, particularly when other key roles such as the Nurse Unit Manager or ward champions had changed. However, it was difficult to convince some key decision makers of the importance of investing in facilitation roles.

*…. they’re not real keen to fund a facilitator because they keep thinking of it as a project officer. I’ve had a meeting with our [senior manager] and tried to explain it to her …. she just doesn’t get it. Like I cannot explain it to her in a way that she understands.* [NF1]

Reflecting on problems related to embedding and sustaining change, novice facilitators reinforced the need to engage key champions, both those with an official role in the implementation project and others more widely in the ward and organizational environment. The experienced facilitators also highlighted an important contribution of facilitating communication between individuals and teams responsible for delivering care.

*We just come across ward after ward that has no mechanism of daily communication between disciplines, at all, and that has no common picture of what they’re trying to achieve […] That’s so much of what the facilitator, you know, tries to do, and give them permission to talk to each other and come up with shared meaning.* (EFs1 & 2]

## Discussion

In this study, we set out to examine how novice facilitators learn and develop knowledge and skills, informed by the i-PARIHS conceptualisation of a mentored journey that is supported by more experienced facilitators. The findings illustrate how, over an 18-month period, the novice facilitators progressively transitioned from novice to more experienced facilitators, consistent with the i-PARIHS proposed facilitation model [25]. They typically changed from a project management or task-focused approach to a more enabling way of working. They established and encouraged interdisciplinary teams to take ownership of implementing the intervention, and gradually moved from a ward facing to an organisational focus. Throughout this journey, novice facilitators displayed learning about themselves, about the facilitator role and the facilitation process.

The support and input from experienced facilitators were critical to supporting the development process of the novice facilitators. The experienced facilitators, building on their previous experience of facilitation and the development of the EWE intervention, used varying strategies and approaches to meet the individual learning and support needs of the novice facilitators. The methods adopted by the experienced facilitators reflect the findings of the earlier study by Ritchie and colleagues, highlighting the use of so-called ‘active’ and ‘participatory’ techniques to transfer implementation facilitation skills [11]. Initially the experienced facilitators used more directive and structured approaches, modelling, teaching, and imparting knowledge. As the program progressed, they provided scaffolding for the novice facilitators’ learning and development by exposure to increasingly complex tasks, and gradually encouraging greater autonomy. The pace and timing of moving from a more to less directive approach varied according to the support needs and confidence of individual novice facilitators. For example, some were accompanied by the experienced facilitator for several implementation work group meetings, whereas others were not. This finding is consistent with Heron’s conceptualisation of the facilitator role, whereby facilitators and the groups they are working with operate along a continuum from directive to non-directive facilitation, with corresponding variations in the level of group autonomy [26]. Other studies of implementation facilitation similarly demonstrate that the process used by external facilitators is fluid, evolves over time and is dependent on the stage of the project [8, 10]. The experienced facilitators recognised and adapted to the needs of the novice facilitator in the current study, providing them with a higher degree of support than they had initially envisaged. As others have noted, the experienced facilitator role was heavily support-oriented [27] and involved emotional support, debriefing, reflecting, problem solving and normalising the challenges faced by novice facilitators.

Alongside the provision of information, coaching and role modelling, the participatory methods identified in Ritchie and colleagues’ research [11] were critically important. Regular, face to face support between experienced facilitators and novice facilitators emerged as an essential element of the facilitation model in practice. It helped decrease novice facilitators’ feelings of isolation, and provided a supportive environment for debriefing, discussion, and reflection on experiences. Structured opportunities for reflection, including reflective writing, have been shown to contribute to facilitator learning and effectiveness [28]. The peer relationships and network between the novice facilitators also emerged as an important element in the development of their knowledge, skills, and confidence. It enabled sharing and learning from each other’s experiences, including coping with negative experiences they encountered and providing a safe space to manage their frustrations. In turn, the novice facilitators encouraged networking and team building in their facilitation role at the project level through identifying and engaging key stakeholders and champions and employing strategies to share ownership of EWE with those involved in implementation. However, this proved time-consuming and challenging due to factors such as staff turnover and other local or organisational priorities.

Reflecting on the facilitation continuum and the facilitation roles proposed in the i-PARIHS framework, the study provides initial support for the idea of a mentored facilitation journey from novice to expert. In particular, the findings highlight the importance of role modelling, adopting a team approach and creating a supportive network, which are key concepts within the i-PARIHS facilitation model. Although i-PARIHS proposes three levels of facilitator, in this study, the focus was mainly on novice and experienced facilitator roles, with some expert facilitator mentoring provided through an implementation steering committee. The novice facilitators demonstrated evidence of transitioning to experienced facilitators within the 18-month timeframe of study. Similarly, the experienced facilitators continued to develop their knowledge and expertise through mentoring and working alongside the novice facilitators. This suggests that the i-PARIHS model of facilitation should be interpreted flexibly depending on the nature and scale of the implementation project, and the existing experience and skills of the facilitators.

The findings provide a preliminary indication of the time and support needed to transition from novice to experienced facilitator, bearing in mind that the four novice facilitators studied were experienced health professionals, purposefully recruited for the role of novice facilitator. Combined with the extensive mentoring and support required from the experienced facilitators, it is evident that facilitated complex interventions in acute care are resource, time, and support intensive. However, as the facilitation network develops and matures, there is potential for it to function as a self-organising community of practice [29], which could off-set some of the resource requirements for mentoring and support.

The interview findings further illuminate the findings of the process evaluation [22], which reports the multiple and diverse activities of the facilitators and the teams they were supporting, and the outcome evaluation [21], which showed significantly reduced delirium and promising improvements in other outcomes, consistent with the program logic. However, further research is required to measure the impact of facilitation and address the question of whether facilitation produces an acceptable return on investment, particularly over the mid to long term in a changing context, reinforcing a need for greater use of health economics evaluation within implementation research [30]. A final observation relates to the gender of the facilitators studied in this research, all of whom were female. There is an emerging interest in sex and gender issues in knowledge translation [31] and this could be an area for further consideration within future research on implementation facilitation.

## Limitations

The current study involved a small sample, with only 2 experienced facilitators and 4 novice facilitators working within a multi-site complex intervention programme. However, this allowed for careful attention to be given to the implementation facilitation across differing contexts. Additionally, no interviews were conducted with the experienced facilitators at time-point 1, although the experienced facilitators were encouraged to reflect on the total period of implementation during their interviews and the data collected were suitably representative and rich. Lastly, data collection consisted of semi-structured interviews only. While this was beneficial in gathering rich descriptions of the facilitators’ experience, we did not objectively assess changes in the novice facilitators’ knowledge, skills, or confidence levels. Future research could seek to implement more objective measures of these elements.

## Conclusion

Within a network of peers and a mentored relationship with experienced facilitators, the novice facilitators followed in this study transitioned along the facilitation continuum to become experienced facilitators. In moving to a more experienced level, novice facilitators developed their knowledge, skills, and confidence in the role, learning both from each other and from more experienced facilitators. Building implementation facilitation capability in this way takes time and requires tailored support and mentorship using a mix of structured and flexible approaches incorporating opportunities for reflection to support individual and group learning.

## Data Availability

The datasets generated and analysed in this research are not publicly available in accordance with local ethics approval but are available from the corresponding author on reasonable request.
